# Microbiome Structure and Mucosal Morphology of Jejunum Appendix and Colon of Rats in Health and Dysbiosis

**DOI:** 10.1007/s00284-023-03224-0

**Published:** 2023-03-06

**Authors:** Chenyi Shao, Xiaobo Song, Lili Wang, Hongying Zhang, Yinhui Liu, Chunhao Wang, Shenmin Chen, Baowei Ren, Shu Wen, Jing Xiao, Li Tang

**Affiliations:** 1 Department of Microecology, College of Basic Medical Sciences, Dalian Medical University, Dalian, China; 2 Department of Medical Biology, Faculty of Health Sciences, The Arctic University of Norway, Tromsø, Norway; 3 Department of Pathology & Forensic Medicine, College of Basic Medical Sciences, Dalian Medical University, Dalian, China; 4 Department of Oral Pathology, College of Stomatology, Dalian Medical University, Dalian, China

## Abstract

Gut microbiota contributes to human health. Plenty of studies demonstrate that antibiotics can disrupt gut ecosystem leading to dysbiosis. Little is known about the microbial variation of appendix and its up/downstream intestine after antibiotic treatment. This study aimed to investigate the microbiome and mucosal morphology of jejunum, appendix, and colon of rats in health and dysbiosis. A rodent model of antibiotic-induced dysbiosis was employed. Microscopy was used to observe mucosal morphological changes. 16S rRNA sequencing was performed for identifying bacterial taxa and microbiome structure. The appendices of dysbiosis were found enlarged and inflated with loose contents. Microscopy revealed the impairment of intestinal epithelial cells. High-throughput sequencing showed the Operational Taxonomic Units changed from 361 ± 33, 634 ± 18, 639 ± 19 in the normal jejunum, appendix, colon to 748 ± 98, 230 ± 11, 253 ± 16 in the disordered segments, respectively. In dysbiosis, *Bacteroidetes* translocated inversely from the colon and appendix (0.26%, 0.23%) to the jejunum (13.87% ± 0.11%); the relative abundance of all intestinal *Enterococcaceae* increased, while *Lactobacillaceae* decreased. Several bacterial clusters were found correlated to the normal appendix, whereas nonspecific clusters correlated to the disordered appendix. In conclusion, species richness and evenness reduced in the disordered appendix and colon; similar microbiome patterns were shared between the appendix and colon regardless of dysbiosis; site-specific bacteria were missing in the disordered appendix. Appendix is likely a transit region involving in upper and lower intestinal microflora modulation. The limitation of this study is all the data were derived from rats. We must be cautious about translating the microbiome results from rats to humans.

## Introduction

The human gastrointestinal tract is home to a large number of microorganisms known as gut microbiome. The distal gut microbiota is mainly composed of strict anaerobes, but also some facultative anaerobes. The quantity of bacteria increases along the gastrointestinal tract, while the stomach has a small number of bacteria due to its acid environment [1]. The composition of gut microbiome can be impacted by many factors, such as intestinal pH; environmental temperature; diet, drug therapy, in addition to the kinship [2]. Extensive studies have shown that the gut microorganisms are involved in human metabolism and nutrition. The gut bacteria can produce a variety of vitamins, synthesize all essential and non-essential amino acids [3], and metabolize non-digestible carbohydrates [4]. The gut microbiota also produces antibacterial compounds, and competes for nutrients and attachment loci in the gut wall to prevent the colonization of pathogens [5]. The gastrointestinal tract is a dynamic microecosystem of which equilibrium is essential to our health. An imbalance in the composition and/or activity of the gut microbiome, which may have negative impacts on health, is referred to as dysbiosis. It may lead to irritable bowel syndrome (IBS) and inflammatory bowel diseases (IBD) [6].

The appendix is a part of the digestive organs located at the junction between the small intestine and large intestine. It was regarded as a functionless vestige from evolutionary history. Nowadays, researchers recognized it as a repository for gut commensal microflora and a part of the immune system [7]. Masses of lymphoid tissues in the appendix enable the adaptation of commensal microflora to the intestinal niches. In addition, the appendix forms immune-mediated biofilms where gut probiotics reside in. Compared to other intestinal regions, the appendix assembles much biofilm as a “hotbed” of intestinal flora [8]. Moreover, its pouch-like structure helps to prevent the loss of gut commensals during diarrhoea [7]. Previous culture-based studies showed that diverse microorganisms, such as *Bacteroides fragilis, Escherichia coli, Pseudomonas aeruginosa, and Peptostreptococcus* species, were frequently isolated from normal and inflammatory appendices [9–11]. In recent years, researchers have started to use gene-sequencing analysis to investigate the microbiome of appendices in health and appendicitis [12–14]. So far, little is known about the ecological variation of appendix and surroundings in the early inflammation, as well as the action of appendix to the gut microbial community during gut dysbiosis.

The present study aimed to compare the microbiome structure and mucosal morphology of the jejunum, appendix and colon of rats in health and dysbiosis. An antibiotic-induced dysbiosis rodent model was established to explore the in situ microbiota in the appendix and its up/downstream intestinal compartments, as well as the pathophysiological changes in the early inflammatory phase.

## Methods

### Rat Handling and In Situ Sampling

Totally, 12 four-week-old Sprague–Dawley (SD) rats were selected and reared adaptively in the laboratory for 1 week. An experimental study was set after allocating the rats randomly into two groups, the experiment group (*n* = 6) and the control group (*n* = 6). The rats in the experiment group were administered orally ceftriaxone sodium at 125 mg/ml dissolved in 0.9% saline solution, 2 ml/day for 14 days to induce gastrointestinal dysbiosis [15]. The rats in the control group were administered orally 0.9% saline solution, 2 ml/day for 14 days in parallel. Then, the rats were sacrificed, and intestinal samples were collected as described below. A 20 cm jejunum segment was sectioned 10 cm beneath the ligament of Treitz. The content was extruded into a 2 ml sterile Eppendorf tube. The appendix segment was cut at the tip of the caecum around the ileocecal junction, and the content was extruded into a sterile tube. A 20 cm colon segment was sectioned 2 cm apart from the end of the ileocecal valve, and the content was extruded into a sterile tube. All the intestinal contents were frozen at −80 °C. The jejunum, appendix and colon segment tissues were fixed in a solution of 10% neutral formalin and 5% glutaraldehyde and stored at 4 °C.

The animal study was reviewed and approved by the Biomedical Ethics Committee, Dalian Medical University. All experiments were performed in accordance with the relevant guidelines and regulations of our Ethical Committee.

### Morphology of Intestinal Segments and Structure of Mucosal Barrier

The gross morphology of the jejunum, appendix and colon and their contents were observed and compared between the two groups.

A 1 cm segment of the jejunum, appendix and colon tissues was embedded in paraffin, and then, the tissue blocks were sectioned at 4 μm. The sections were stained with haematoxylin and eosin and observed with an optical microscope (DP73, Olympus, Tokyo, Japan).

Tissue blocks of 1 mm × 3 mm from each segment were placed in precooled 2.5% glutaraldehyde for 2 h, rinsed with 0.1 M phosphate buffer (pH 7.2) for 15–20 min (4 °C) three times, fixed with 1% osmium tetroxide for 2 h (4 °C), rinsed with 0.1 M phosphate buffer for 5 min (4 °C) three times, washed with double-distilled water and dried. The blocks were soaked and embedded in methyloxirane and the embedded liquid and then sectioned into semithin section smears. The sections were stained, dried, dyed (toluidine blue), washed, cleared and sealed and observed under a transmission electron microscope (JEM-2000EX, JEOL, Tokyo, Japan). The experiments were performed in triplicate.

For evaluation of the severity of inflammation, five randomly selected fields in each section (magnification × 100) were inspected and graded by a pathologist blinded to the group allocation. Scores were generated according to the criteria of the modified scale of Bobin-Dubigeon et al. [16]. After the 5 fields were graded, the mean score was calculated for each section and is expressed as the histological score.

### DNA Isolation and 16S rRNA Gene Sequencing

An E.Z.N.A. Stool DNA kit (Omega Bio-Tek, Inc., Norcross, GA, United States) was used for whole DNA extraction from the stool. The DNA concentration was measured with Qubit 2.0 (Invitrogen, Carlsbad, CA, United States). Polymerase chain reaction (PCR) was applied to the bulk DNA with the barcoded primers 341F (5' -CCTAYGGGRBGCASCAG- 3') and 806R (5' -GGACTACNNGGGTATCTAAT-3'), which cover the V3–V4 regions of the 16S rRNA gene [17]. PCR reactions were performed on an ABI GeneAmp 9700 PCR system (Applied Biosystems, Foster City, CA, United States).

The 16S amplicons were purified with a GeneJET PCR Purification Kit (Thermo Fisher Scientific, Waltham, MA, United States). A DNA library was constructed by using the Ion Plus Fragment Library Kit 48 rxns (Thermo Fisher Scientific, Waltham, MA, United States). After the library was quantified with a Qubit fluorometer (Qubit 3.0, Invitrogen, Carlsbad, CA, United States) and qualified, it was sequenced by an Ion S5 XL system (Thermo Fisher Scientific, Waltham, MA, United States).

### Bioinformatic and Statistical Analysis

Quality filtering was performed on the raw reads to obtain high-quality clean reads. According to Cutadapt (v1.9.1) [18] (http://cutadapt.readthedocs.io/en/stable/), the reads were compared with the GOLD reference database (http://drive5.com/uchime/uchime_download.html) with the UCHIME algorithm (http://www.drive5.com/usearch/manual/uchime_algo.html) to detect and remove chimaeric sequences to obtain clean reads [19, 20].

Sequence analysis was performed with UPARSE software (Uparse v7.0.1001) (http://drive5.com/uparse/) [21]. Sequences with ≥ 97% similarity were assigned to the same operational taxonomic units (OTUs). Representative sequences for each OTU were screened for further annotation. For each representative sequence, the SSU rRNA [22] database of Silva (http://www.arb-silva.de/) [23] was used based on the Mothur algorithm to annotate taxonomic information (set threshold from 0.8 to 1). For determination of the phylogenetic relationships of different OTUs and the difference in the dominant species in different samples (groups), multiple sequence alignments were conducted using MUSCLE (http://www.drive5.com/muscle/) Software (v3.8.31) [24]. OTUs abundance information was normalized using a standard sequence number corresponding to the sample with the fewest sequences. Subsequent analyses of alpha diversity and beta diversity were all performed based on these output normalized data.

Data are expressed as the mean ± standard error of the mean. Alpha diversity was applied to analyse the complexity of species diversity for a sample through 2 indices, observed species and Chao1 indices. Both of these indices in our samples were calculated with QIIME (Version 1.7.0). The Wilcox test in the agricolae package of R software (Version 2.15.3) was used to analyse the between-group difference in alpha diversity. Beta diversity was applied with Permutational multivariate analysis of variance (Adonis) analysis and the nonmetric multidimensional scaling (NMDS) analysis. NMDS analysis was based on Bray–Curtis dissimilarity and performed by the vegan software package of R software. The correlation between microbiome taxa and rosuvastatin effectiveness was assessed using linear discriminant analysis (LDA) effect size (LEfSe) at various taxonomic ranks [25]. An LDA score greater than 4.0 was defined as significant by default. LEfSe data were analysed using R software, and analysis of variance (ANOVA) was used to identify the relative abundance differences between groups. Tukey’s test was applied to perform post hoc tests, with *P* < 0.05 considered a significant difference. PICRUSt2 was performed using the OmicStudio Analysis (https://www.omicstudio.cn/analysis/) to predict the functional profiles of intestinal microbiome. T-test was used for analysing the OTU abundance from the same gut segment between the two groups OmicStudio tools (https://www.omicstudio.cn/tool) was utilized for statistical analyses and visualization of the identified pathways. R software was used for permutational multivariate analysis of variance (Adonis) to analyse the between-group differences in beta diversity. Group comparisons of histological scores were statistically analysed using independent-samples t-tests (SPSS 19.0). Statistical significance was accepted at *P* < 0.05. Twenty-five appendicitis-associated taxa reported previously (Table 1), such as *Actinobacteria*, *Proteobacteria*, and *Fusobacteria*, were analysed from our samples with/without dysbiosis [13, 26–29].

**Table 1:** Appendicitis-associated taxa reported in previous studies

Phylum	Genus	Species
*Firmicutes* *Bacteroidetes* *Actinobacteria* *Proteobacteria* *Fusobacteria*	*Streptococcus* *Gemella* *Bacteroides* *Faecalibacterium* *Proteus* *Fusobacterium* *Rhizobium* *Porphyromonas* *Mogibacterium* *Prevotella* *Bilophila* *Dialister* *Anaerofilum* *Bergeyella* *Peptostreptococcus* *Fusibacter* *Parvimonas*	*Escherichia coli* *Bacteroides fragilis* *Porphyromonas endodontalis*

## Results

### Intestinal Morphology and Mucosal Structure from the Appendix, Jejunum and Colon of the Rats w/o Dysbiosis

**Fig. 1:**
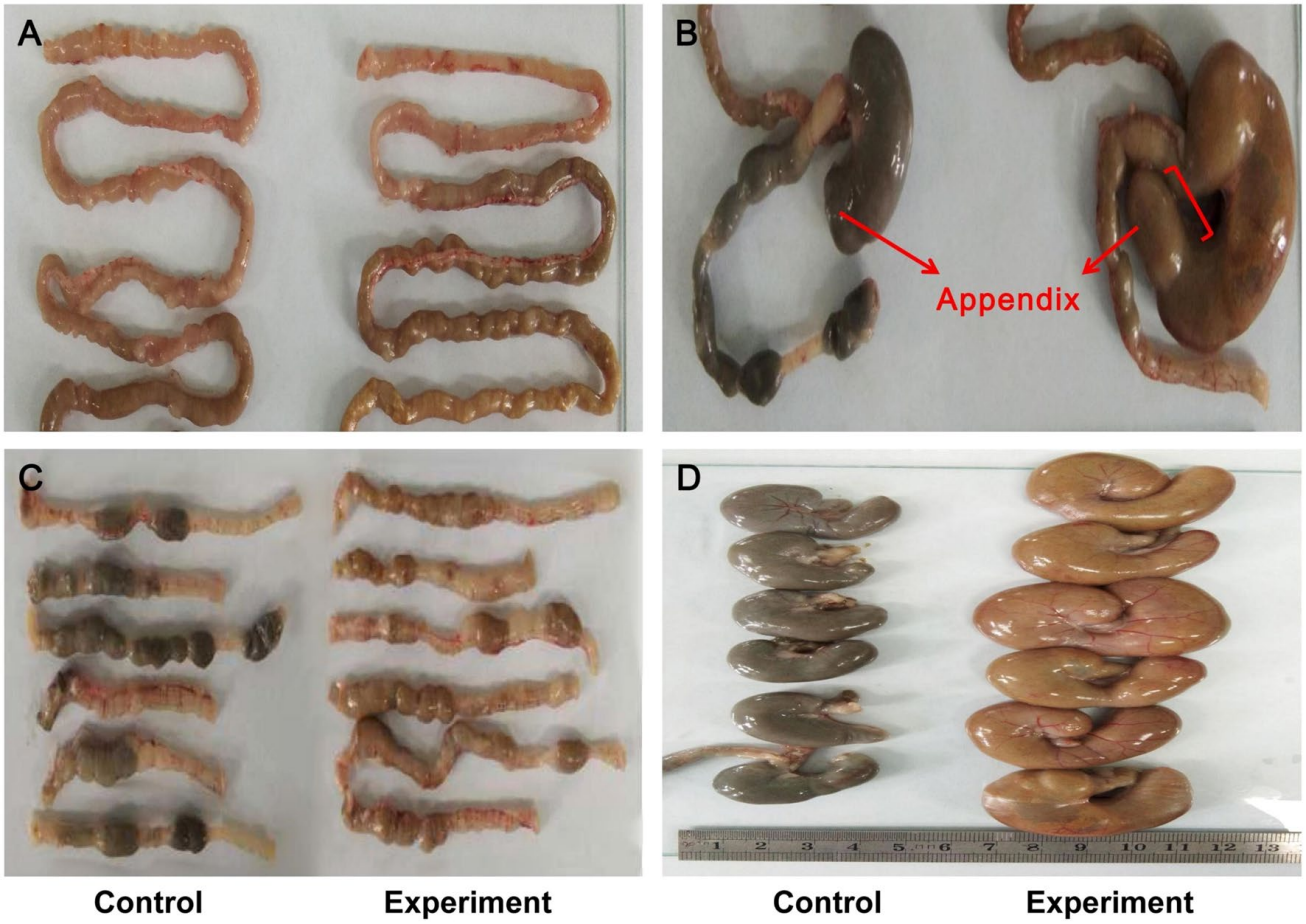
The gross morphologies of jejunum, appendix and colon were compared between control and experiment group. (**A**) Experiment jejunum had no obvious changes; (**B**, **D**) Experiment appendices became enlarged and inflated with loose and light-coloured contents; (**C**) Experiment colon contents were loose and light coloured (Color figure online)

The gross morphology of the three intestinal segments was compared between the two groups: experiment group (referred to as ceftriaxone-treated group) and control group (referred to as placebo group). In the experiment group, the jejunum had no obvious changes (Fig. 1A); the appendices became enlarged and inflated with increasing, loose and light-coloured contents (Fig. 1B, D); the colon contents were also loose and light coloured (Fig. 1C). The morphological variations suggested abnormal absorption and/or exudation arose in the appendix and colon.

**Fig. 2:**
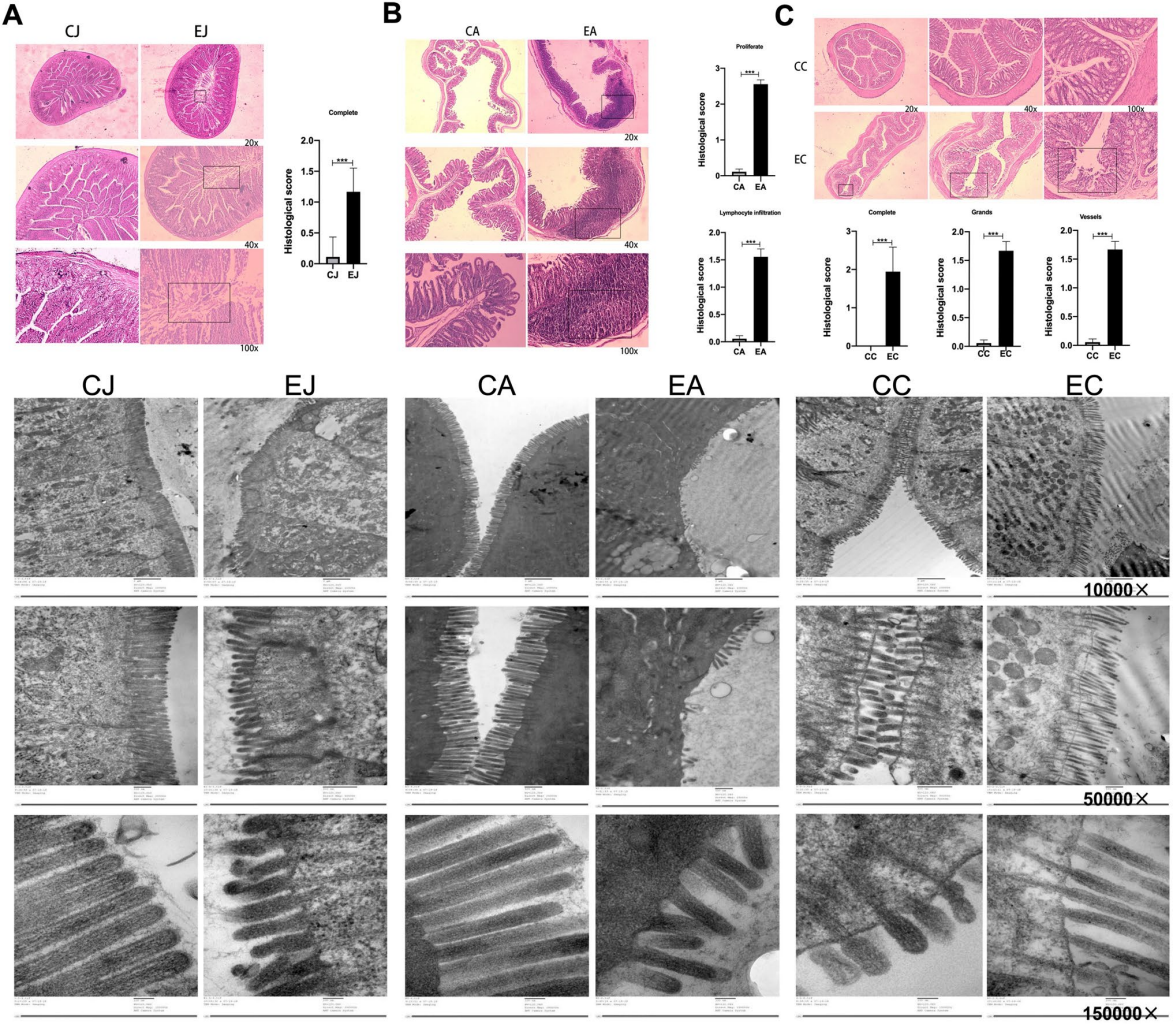
Representative histological sections of (**A**) jejunum, (**B**) appendix and (**C**) colon under microscope (above: light microscopy, down: electron microscopy) and histological scores (*significant difference with *P*< 0.05). *CJ* control jejunum, *CA* control appendix, *CC* control colon, *EJ* experiment jejunum, *EA* experiment appendix, *EC* experiment colon

Light microscopy reveals the mucosal morphology of the jejunum (Fig. 2A), appendix (Fig. 2B) and colon (Fig. 2C) in both groups. In the control group, the colonic surface of the mucosa was smooth, and all mucosal epithelial cells were intact. In the experiment group, more than half of the disordered jejunal epithelium was incomplete, and a few epithelial cells fell into the intestinal cavity. The lumen of the ceftriaxone-treated appendix was narrower than that of the control appendix. The local lymph nodules proliferated in the antibiotic-treated appendix, and the adjacent fibrous connective tissue also proliferated. The colonic mucosal layer became thinner; the mucosal epithelium was incomplete, and the cells fell into the intestinal cavity; most of the glands disappeared; the vessels of the lamina propria and submucosa were dilatable and congested; and fibrous connective tissue obviously proliferated. In the rest of the lamina propria and submucosa, the blood vessels were dilated, and hyperaemia and oedema with slight hyperplasia of fibrous connective tissue were observed. The graphs of the histological scores illustrate the differences in the intestinal sections between the groups.

Transmission electron microscopy reveals the epithelium ultrastructure of the jejunum, appendix and colon (Fig. 2). In the experiment group, the jejunum epithelial cells were oedematous, the mitochondria were swollen, and the microvilli were disorganized; the microvilli in the appendix became short, ruptured and dispersed; the colonic epithelial cells were oedematous, and the microvilli were sparse. All the above impairments indicated that the ceftriaxone treated appendix and the up/downstream intestinal compartments were in the inflammatory responses.

### Diversity and Abundance of Microbiome from the Jejunum, Appendix, and Colon of the Rats w/o Dysbiosis

Based on high-throughput sequencing, 16S rRNA sequence data were processed and analysed with bioinformatic and statistical packages. The numbers of OTUs represent species richness. The mean number of reads was 55576.19 (with two decimal places), and the range was 46836 to 66648.

**Fig. 3:**
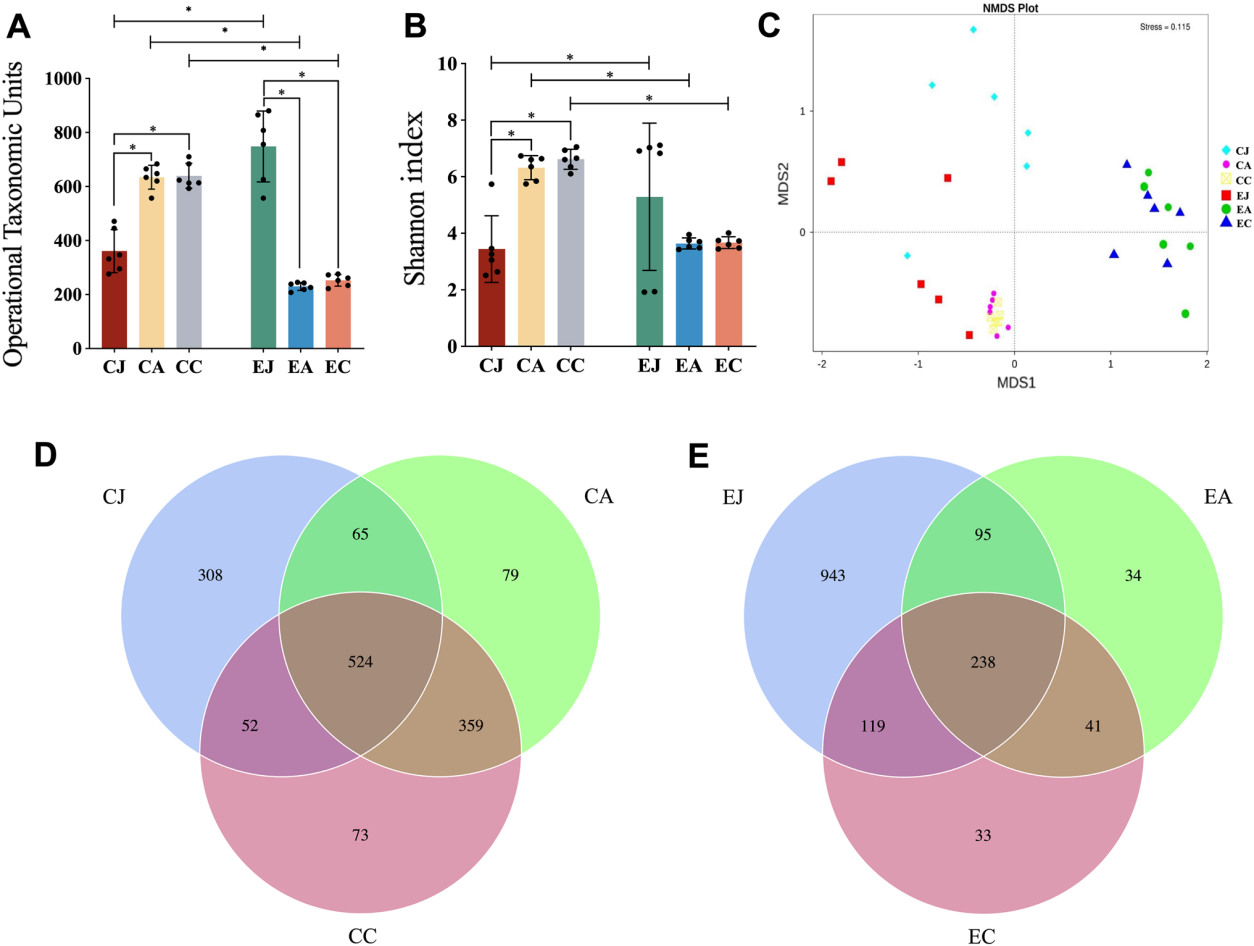
Metagenomic analysis of the gut microbiome of control and experiment group. (**A**) observed OTUs; (**B**) Shannon diversity; (**C**) NMDS is analysed based on Bray–Curtis distance; (**D**) Venn diagram showing the number of OTUs specific and common to the three sites in the control group; (**E**) Venn diagram showing the number of OTUs specific and common to the three sites in the experiment group. *CJ* control jejunum; *CA* control appendix; *CC* control colon; *EJ* experiment jejunum; *EA* experiment appendix; *EC* experiment colon

In the control group, the microbiome OTUs numbers were 361±33, 634 ± 18 and 639 ± 19 in the jejunum, appendix and colon, respectively (Fig. 3A). The species richness, abundance and evenness were high in the appendix and colon compared to the jejunum (Fig. 3A, B). The number of common OTUs was 524 in the three segments (Fig. 3D), and the number of distinct OTUs was 308 in the jejunum, 79 in the appendix and 73 in the colon. The number of OTUs shared by the jejunum and appendix was 65, the number shared by the jejunum and colon was 52, and the number shared by the appendix and colon was 359. More OTUs were shared by the appendix and the colon.

In the experiment group, the numbers of OTUs were 748 ± 98, 230 ± 11 and 253 ± 16 in the jejunum, appendix, and colon, respectively (Fig. 3A). The species richness, abundance and distribution evenness were high in the jejunum. The species diversity and evenness fell in the appendix and colon (Fig. 3A, B). The number of common OTUs in the jejunum, appendix and colon was 238 (Fig. 3E), and the number of distinct OTUs was 943, 34 and 33 in the jejunum, appendix and colon, respectively. The number of OTUs shared by the jejunum and appendix was 95, by the jejunum and colon was 119, and by the appendix and colon was 41. Adonis analysis (Table 2) and Non-metric multidimensional scaling (NMDS) analysis (Fig. 3C) showed that the microbiome structure of the jejunum was different from that of the appendix and colon. Similar microbial patterns were identified in the appendix and the colon in both groups.

**Table 2:** Adonis analyse of microbiome from jejunum, appendix and colon

Vs Group	*R*^2^	*P* value
CJ–CA	0.34311	0.001
CJ–CC	0.40668	0.001
CA–CC	0.08208	NS
EJ–EA	0.35951	NS
EJ–EC	0.32536	0.001389
EA–EC	0.03698	NS
CJ–EJ	0.36004	0.012
CA–EA	0.49038	0.012
CC–EC	0.49475	0.001

*CJ* control jejunum, *CA* control appendix, *CC* control colon, *EJ* experiment jejunum, *EA* experiment appendix, *EC* experiment colon, *NS* no significant

### Composition of the Microbiome from the Appendix, Jejunum and Colon of the Rats w/o Dysbiosis

The gut regional microbiome was classified at the phylum, family and genus levels. At each taxon level, the top ten most abundant bacteria were selected for comparative analysis (detailed data are shown in Table 3).

**Table 3:** Relative abundance of microbiome from jejunum, appendix, and colon at phylum, family and genus level

Taxon	CJ (%)	CA (%)	CC (%)	EJ (%)	EA (%)	EC (%)
Phylum:* Firmicutes*	80.37 ± 0.28	73.90 ± 0.11	56.87 ± 0.14	50.05 ± 0.39	79.46 ± 0.22	83.40 ± 0.14
*Bacteroidetes*	10.85 ± 0.25	22.09 ± 0.13	40.23 ± 0.14^#θ^	13.87 ± 0.11	0.23 ± 0.00	0.26 ± 0.00
*Tenericutes*	5.56 ± 0.08	0.27 ± 0.00^β^	0.22 ± 0.00^θ^	0.98 ± 0.01*'	18.65 ± 0.21	12.74 ± 0.12
*Cyanobacteria*	0.17 ± 0.00^α^	0.19 ± 0.00	0.45 ± 0.00	9.64 ± 0.08*'	0.02 ± 0.00	0.02 ± 0.00^#'^
*Actinobacteria*	1.78 ± 0.00^α^	1.24 ± 0.01	0.64 ± 0.00	6.70 ± 0.05*'	0.07 ± 0.00	0.07 ± 0.00^#'^
*Fusobacteria*	0.01 ± 0.00^α^	0.00 ± 0.00	0.01 ± 0.00	1.06 ± 0.02*'	0.00 ± 0.00	0.00 ± 0.00^#'^
*Proteobacteria*	1.15 ± 0.01	2.17 ± 0.01	1.52 ± 0.01	15.76 ± 0.13	0.09 ± 0.00	0.11 ± 0.00
*Deinococcus-Thermus*	0.01 ± 0.00^α^	0.00 ± 0.00	0.00 ± 0.00	0.56 ± 0.00*'	0.01 ± 0.00	0.00 ± 0.00^#'^
*SR1 (Absconditabacteria)*	0.00 ± 0.00^α^	0.00 ± 0.00	0.00 ± 0.00	0.29 ± 0.00*'	0.00 ± 0.00	0.00 ± 0.00^#'^
*Euryarchaeota*	0.01 ± 0.00^α^	0.00 ± 0.00	0.00 ± 0.00	0.16 ± 0.00*'	0.00 ± 0.00	0.00 ± 0.00^#'^
Family:* Enterococcaceae*	0.28 ± 0.00^α^	0.17 ± 0.00	0.15 ± 0.00	35.14 ± 0.48	16.84 ± 0.07	21.13 ± 0.10
*Lactobacillaceae*	68.69 ± 0.28*^α^	22.40 ± 0.15	11.24 ± 0.07^#^	2.13 ± 0.01	0.59 ± 0.00	1.60 ± 0.02
*Clostridiales_vadin* *BB60_group*	0.17 ± 0.00	0.37 ± 0.00^β^	0.36 ± 0.00^θ^	0.36 ± 0.00*'	46.97 ± 0.29	42.04 ± 0.28^#'^
*Bacteroidales_S24-7_group*	10.55 ± 0.25	18.16 ± 0.10	29.47 ± 0.04^#θ^	2.26 ± 0.01	0.17 ± 0.00	0.17 ± 0.00
*Anaeroplasmatacee*	0.06 ± 0.00	0.09 ± 0.00^β^	0.09 ± 0.00^θ^	0.10 ± 0.00*'	18.42 ± 0.21	11.75 ± 0.12
*Lachnospiraceae*	0.51 ± 0.00*	29.59 ± 0.09^β^	23.53 ± 0.11^#θ^	2.59 ± 0.01	0.21 ± 0.00	0.25 ± 0.00
*Erysipelotrichaceae*	6.83 ± 0.09	1.14 ± 0.01	1.41 ± 0.02	0.94 ± 0.00	10.05 ± 0.15	13.96 ± 0.22
*Prevotellaceae*	0.11 ± 0.00	3.42 ± 0.03	9.63 ± 0.13^#^	3.36 ± 0.03	0.02 ± 0.00	0.03 ± 0.00
*Ruminococcaceae*	0.27 ± 0.00*	18.49 ± 0.08^β^	18.25 ± 0.06^#θ^	1.78 ± 0.00	0.15 ± 0.00	0.19 ± 0.00
*Mycoplasmataceae*	5.50 ± 0.08*	0.04 ± 0.00	0.04 ± 0.00^#^	0.84 ± 0.01	0.23 ± 0.00	0.99 ± 0.00
Genus:* Enterococcus*	0.28 ± 0.00	0.17 ± 0.00^β^	0.15 ± 0.00	35.11 ± 0.48	16.83 ± 0.07	21.13 ± 0.10
*Lactobacillus*	68.69 ± 0.28*^α^	22.40 ± 0.15	11.24 ± 0.07^#^	2.10 ± 0.01	0.59 ± 0.00	1.60 ± 0.02
*Anaeroplasma*	0.06 ± 0.00	0.09 ± 0.00	0.09 ± 0.00^θ^	0.14 ± 0.00*'	16.41 ± 0.21	12.47 ± 0.12
*Erysipelotrichaceae_UCG-004*	0.07 ± 0.00	0.06 ± 0.00	0.05 ± 0.00^θ^	0.08 ± 0.00	9.99 ± 0.15	13.89 ± 0.22
*Prevotella_9*	0.06 ± 0.00	1.29 ± 0.02	4.79 ± 0.09	0.44 ± 0.00	0.01 ± 0.00	0.02 ± 0.00
*Mycoplasma*	5.50 ± 0.08*^α^	0.04 ± 0.00	0.04 ± 0.00^#^	0.84 ± 0.01	0.23 ± 0.00	0.99 ± 0.00
*Turicibacter*	5.02 ± 0.08	0.27 ± 0.00	0.49 ± 0.00	0.27 ± 0.00	0.03 ± 0.00	0.04 ± 0.00
*unidentified_Chloroplast*	0.15 ± 0.00	0.02 ± 0.00^β^	0.02 ± 0.00	9.57 ± 0.08*'	0.02 ± 0.00	0.02 ± 0.00^#'^
*Bacteroides*	0.07 ± 0.00	0.23 ± 0.00^β^	0.49 ± 0.00	6.56 ± 0.06*'	0.01 ± 0.00	0.04 ± 0.00^#'^
*Lachnospiraceae_N* *K4A136_group*	0.07 ± 0.00*^α^	7.13 ± 0.03^β^	5.38 ± 0.03^#θ^	0.39 ± 0.00	0.05 ± 0.00	0.06 ± 0.00

Data are expressed as mean ± standard error of the mean

*CJ* control jejunum, *CA* control appendix, *CC* control colon, *EJ* experiment jejunum, *EA* experiment appendix, *EC* experiment colon

Significant difference with *P* < 0.05 when comparing the microbiome between ^*^CJ and CA; ^#^CJ and CC; *' EJ and EA; ^#'^ EJ and EC; ^α^ CJ and EJ; ^β^ CA and EA; ^θ^ CC and EC

**Fig. 4:**
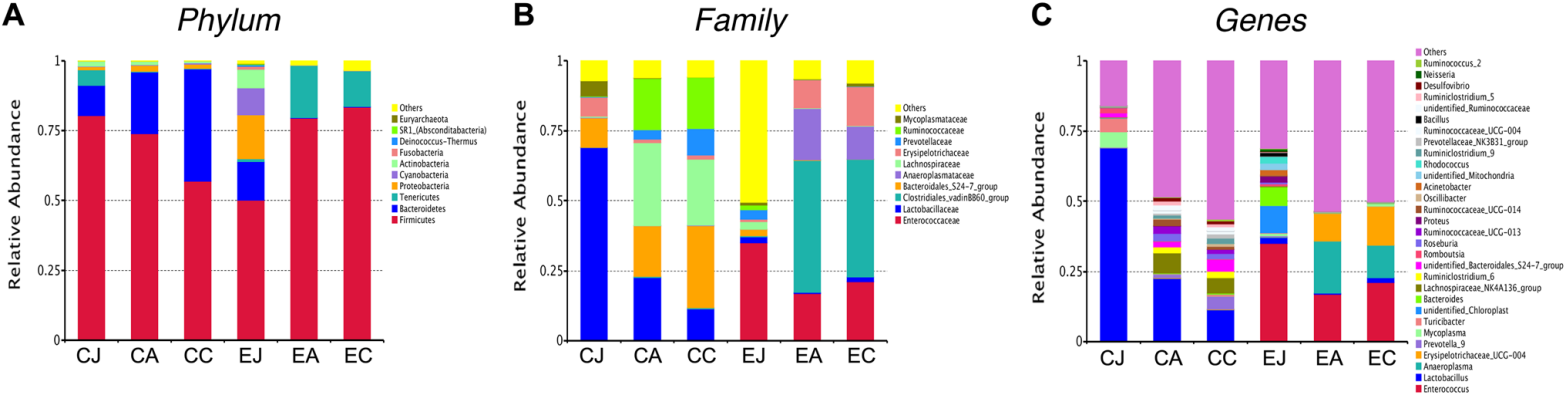
Predominant intestinal microbiome and their relative abundance. (**A**) top 10 microbiome at phylum level; (**B**) top microbiome 10 at family level; (**C**) top 30 microbiome at genus level. *CJ* control jejunum; *CA* control appendix; *CC* control colon; *EJ* experiment jejunum; *EA* experiment appendix; *EC* experiment colon

At the phylum level, the composition of the microbiome was different among the three segments and between the two groups (Fig. 4A). In the control group, the relative abundance of most phyla decreased down from the jejunum to the colon. *Firmicutes* and *Bacteroidetes* were the main constituent microbiomes from all segments. *Firmicutes* showed a declining relative abundance (80.37% ± 0.28%, 73.90% ± 0.11% and 56.87% ± 0.14%) from the jejunum down to the colon. *Bacteroidetes* showed an increasing relative abundance (10.85% ± 0.25%, 22.09% ± 0.13% and 40.23% ± 0.14%) along the intestinal tract. *Fusobacteria*, *Deinococcus-Thermus*, and *Euryarchaeota* were barely detected (detailed data shown in Table 3). In the experiment group, the *Firmicutes* and *Bacteroidetes* were opposite to those in the control group. *Firmicutes* showed an increasing relative abundance (50.05% ± 0.39%, 79.46% ± 0.22% and 83.40% ± 0.14%) from the jejunum down to the colon. *Bacteroidetes* showed a declining relative abundance (13.87% ± 0.11%, 0.23% and 0.26%). *Tenericutes* also varied compared to that in the control group, with the relative abundance being high in the appendix and colon but low in the jejunum. *Proteobacteria*, *Cyanobacteria* and *Actinobacteria*, like *Bacteroidetes*, were highly abundant in the jejunum but almost undetectable in the appendix and colon. Other phyla were detected in a low relative abundance from the three intestinal segments (detailed data shown in Table 3).

At the family level, the relative abundances of the microbiome were compared between the segments and the groups (Fig. 4B). In the control group, *Lactobacillaceae* was the main constituent flora from the jejunum to the colon at relative abundances of 68.69% ± 0.28%, 22.40% ± 0.15% and 11.24% ± 0.07%. *Enterococcaceae* accounted for a very small proportion of the gut microbiota (0.28% ± 0.00%, 0.17% ± 0.00%, 0.15% ± 0.00%). Other families showed higher relative abundances in the appendix and colon than in the jejunum (detailed data shown in Table 3). In the experiment group, *Enterococcaceae* increased in the jejunum, appendix and colon (35.14% ± 0.48%, 16.84% ± 0.07% and 21.13% ± 0.10%), whereas *Lactobacillaceae* decreased in all three segments (2.13% ± 0.01%, 0.59% ± 0.00% and 1.60% ± 0.02%). A dramatic increase of *Clostridiales_vadinBB60_group* was identified in the appendix and colon (46.97% ± 0.29% and 42.04% ± 0.28%). *Erysipelotrichaceae* and *Mycoplasmataceae* were abundant in the jejunum (6.83% ± 0.09%, 5.50% ± 0.08%) but scant in the appendix (1.14% ± 0.01%, 0.04% ± 0.00%) and the colon (1.41% ± 0.02%, 0.04% ± 0.00%) despite of dysbiosis. *Anaeroplasmataceae* and *Erysipelotrichaceae* increased in the appendix and colon. *Prevotellaceae, Lachnospiraceae, Bacteroidales_S24-7_group* and *Ruminococcaceae* were found only in the jejunum at low relative abundances (detailed data shown in Table 3).

At the genus level, statistical analysis was performed to compare the top 30 genera within and between the groups (Fig. 4C). In the control group, the top 30 genera accounted for over 85% of the bacteria in the jejunum, 52% in the appendix and 44% in the colon. *Lactobacillus* was the dominant genus in all segments (68.69% ± 0.28%, 22.40% ± 0.15% and 11.24% ± 0.07%). *Enterococcus* was rare (0.28% ± 0.00%, 0.17% ± 0.00% and 0.15% ± 0.00%). The majority of these genera, except *Lachnospiraceae_NK4A136_group* and *Prevotella_9,* showed higher relative abundances in the jejunum than in the appendix and colon (detailed data shown in Table 3). In the experiment group, *Enterococcus* appeared to be the main genus from the jejunum to the colon (35.11% ± 0.48%, 16.83% ± 0.07% and 21.13% ± 0.10%), while *Lactobacillus* reduced dramatically in the disordered guts (2.10% ± 0.01%, 0.59% ± 0.00% and 1.60% ± 0.02%). *Bacteroides* and *unidentified_Chloroplast* were detected in the jejunum at relative abundances of 6.56% ± 0.06% and 9.57% ± 0.08% and were almost undetectable in the appendix and colon. *Anaeroplasma* and *Erysipelotrichaceae_UCG-004* were barely detected in the jejunum but were highly detected in the appendix (16.41% ± 0.21%, 9.99% ± 0.15%) and the colon (12.47% ± 0.12%, 13.89% ± 0.22%) (detailed data shown in Table 3).

### Intergroup/Intragroup Analysis of Microbial Clusters from Different Intestinal Sites

**Fig. 5:**
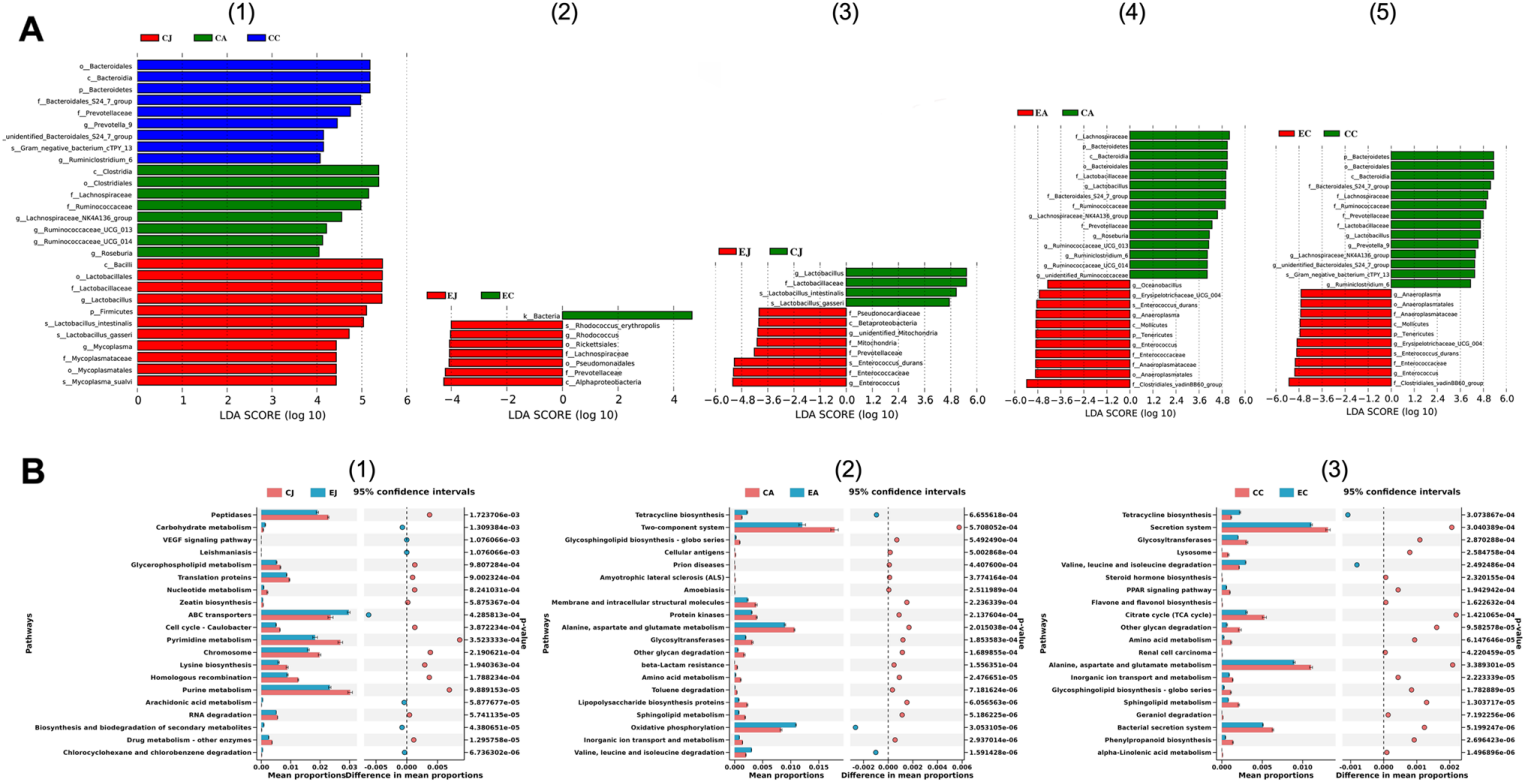
LEfSe analysis (**A**) and functional profiles (**B**) of intestinal microbiome. (A1) LDA scores of the segmental microbiome of control group; (A2) LDA scores of the segmental microbiome of experiment group; (A3) LDA scores of jejunum microbiome between the two groups; (A4) LDA scores of appendix microbiome between the two groups; (A5) LDA scores of colon microbiome between the two groups (LDA score threshold set at 4); (B1) 20 most different functional pathways between CJ and EJ; (B2) 20 most different functional pathways between CA and EA; (B3) 20 most different functional pathways between CC and EC. *CJ* control jejunum; *CA* control appendix; *CC* control colon; *EJ* experiment jejunum; *EA* experiment appendix; *EC* experiment colon

To compare the microbial clusters from the different intestinal sites within and between groups, we used LEfSe software to determine the metagenomic differences of the OTUs derived from 16S rRNA sequences from the jejunum, appendix and colon. In the control group, a total of 28 microbial clusters showed site-specific abundances (LDA value > 4.0), including 11 in the jejunum, eight in the appendix, and nine in the colon (Fig. 5A1). In the experiment group, eight microbial clusters showed site-specific abundances (LDA value > 4.0), including seven in the jejunum and one in the colon (Fig. 5A2). No site-specific clusters were found in the disordered appendix. Comparing the two groups, we found 12 different bacterial clusters in the jejunum (Fig. 5A3), 26 different clusters in the appendix (Fig. 5A4) and 24 different clusters in the colon (Fig. 5A5). Of the jejunum, *Enterococcus* and *Lactobacillus* varied greatly between the healthy and disordered jejunum. Of the appendix and the colon, *Lachnospiraceae*, *Bacteroidales_S24_7_group*, and *Clostridiales_vadinBB60_group* varied greatly between the two groups. PICRUSt2 analysis was used to predict metagenomic functions associated with bacterial communities based on 16S rRNA sequencing data. At KEGG levels 3, there are 56 different functional pathways found in the jejunum, and on 100 and 105 functional pathways found in the appendix and colon between the two groups. Figure 5B shows the 20 functional pathways with the highest significant difference in the jejunum, appendix, and colon between the two groups.

### Appendicitis-Associated Taxa Identified from the Jejunum, Appendix and Colon of the Rats w/o Dysbiosis

Former researchers reported that 25 bacterial taxa, five phyla, 17 genus and three species were commonly associated with appendicitis (Table 1) [13, 26–29]. We analysed these appendicitis-associated taxa in our samples and found that some microbes had special preferences for the different intestinal sites. At the phylum level, the relative abundance of *Actinobacteria* was 1.78% ± 0.00%, 1.24% ± 0.01% and 0.64% ± 0.00% in the control jejunum, appendix and colon, respectively, but changed to 6.70% ± 0.05%, 0.07% ± 0.00% and 0.07% ± 0.00% in the experiment jejunum, appendix and colon, respectively. The relative abundances of *Proteobacteria* were 1.15% ± 0.01%, 2.17% ± 0.01% and 1.52% ± 0.01% from the control jejunum, appendix and colon, respectively, but changed to 15.76% ± 0.13%, 0.09% ± 0.00% and 0.11% ± 0.00% in the experiment group. *Fusobacteria* was barely detected in the control group but increased to 1.06% ± 0.02% in the experiment jejunum. At the genus level, the relative abundances of *Fusobacterium*, *Streptococcus*, *Porphyromonas* and *Proteus* were increased in the experiment jejunum. Other genus showed no significant change in the experiment group. At the species level, *E. coli* and *B. fragilis* increased in the experiment jejunum.

## Discussion

Trillions of microorganisms inhabit the gastrointestinal tract of complex multicellular animals and humans. They play a vital role in dietary metabolism and digestive system health. Due to the development of high-throughput sequencing technology, the impact of gut flora on human health and disease has been explored in recent years. The appendix is an organ of the digestive tract where bulk of commensals inhabit. Some studies have revealed that appendicitis is precipitated by luminal obstruction and subsequent microbial overgrowth [30]. The present study investigated the in situ microbiome and mucosal morphology of rat jejunum, appendix and colon with/without antibiotic-induced dysbiosis. We intended to gain an insight into the ecology of appendix and its up/downstream intestine as well as the protective role of appendix to gut microbial community.

In this experiment, a rodent model of antibiotic-induced dysbiosis was established in order to imitate human dysbiosis and to obtain gut microbiome in situ. Ceftriaxone is a third-generation cephalosporin with a wide spectrum of activity against gram-negative bacilli and most gram-positive bacteria. The present study shows that oral antibiotic administration changes the microbial community and mucosal morphology of the appendix and the up/downstream intestinal compartments. Our metagenomic data revealed that the species richness and evenness were higher in the jejunum than in the appendix and colon of dysbiosis. The reason for this may be that the peristalsis speed of the jejunum is faster than that of the appendix and colon. Ceftriaxone remained longer in the large intestine than the other organs so that it enhanced the inhibitory effect on the gut microbiota. On the other hand, some of the flora that are sensitive to prolonged antibiotic effects might shift retrogradely into the upper intestine. For example, *Bacteroides* translocated from the colon to the jejunum during dysbiosis. Other bacteria that rely on the end products from these bacteria possibly migrate together to ingest carbon and nitrogen sources [31]. Thus, the community diversity increases in the upper bowel during dysbiosis. Another interesting finding is that the appendix and colon share similar microbiome pattern regardless of dysbiosis. This reflects the similar ecosystem of appendix and colon despite of the challenges from antibiotics or inflammation.

*Firmicutes* and *Bacteroidetes* were recovered to be the most dominant phyla among the gut microflora in this study. The relative abundance of *Firmicutes* was decreasing from the upper to the lower intestine of the healthy rats, but this tendency reversed in the disordered rats. Nevertheless, the relative abundance of *Firmicutes* remained stable in the appendix. *Firmicutes* is a phylum of bacteria, most of which have gram-positive cell wall structures. More than 274 genera were assigned to the *Firmicutes* phylum. Notable genera of *Firmicutes* are *Enterococcus* and *Lactobacillus* etc. The main function of *Firmicutes* is to hydrolyse carbohydrates and proteins in the intestine [32]. A sufficient level of *Firmicutes* in the healthy jejunum complies with the function of the small intestine, where most chemical digestion of carbohydrates, proteins and fats occurs. When the intestinal flora was disrupted, the amount of *Firmicutes* dropped in the jejunum and increased in the colon. *Bacteroidetes* distributed inversely to *Firmicutes* in the guts of both groups. *Bacteroidetes* are primary colonizers of the colon that involve in the metabolism of steroids, polysaccharides and bile acids, as well as polysaccharide utilization and protein synthesis [33]. A previous work found the same variation in microbiota among obese children that *Firmicutes* increased and *Bacteroidetes* decreased in the colon [34]. Schade demonstrated that the cell wall glycopolymers of *Firmicutes* could influence host microbe interactions through the modulation of bacterial colonization [35].

*Lactobacillaceae* and *Enterococcaceae* are two families under the phylum *Firmicutes*. In the healthy jejunum, *Lactobacillaceae* abundance was high, while the relative abundance of *Enterococcaceae* was extremely low, less than 0.3%. The jejunum favours the survival and reproduction of *Lactobacillaceae* because the pH value is around 6.1 in the proximal jejunum and 7.3 in the terminal ileum [36]. When the intestinal flora was disrupted, *Enterococcaceae* became predominant in the jejunum, and *Lactobacillaceae* decreased significantly. It is likely that *Enterococcaceae* and *Lactobacillaceae* react adversely to each other. *Lactobacillaceae* is a group of gram-positive, facultative anaerobes that can convert sugars to lactic acid. They are the predominant gut microbiota and the most common probiotics added to foods, and it has antimicrobial potential against pathogens [37].

To compare the in situ microbial composition, we used LEfSe analysis and species abundance clustering to estimate the beta diversity of the bacterial OTUs. In a normal intestinal ecosystem, the distribution of microbiota reflects the tissue tropism of different intestinal parts. *Lachnospiraceae* was found abundant in the control appendix, whose species showed a negative correlation with the development of obesity and diabetes [38]. *Ruminococcaceae*, *Desulfovibrionaceae* and *Coriobacteriaceae* were also abundant in the control appendix. *Ruminococcaceae* is the member of commensal bacteria of the caecum and colon [39], which can degrade various polysaccharides and fibres [40]. *Desulfovibrionaceae* is a group of sulphate-reducing bacteria that can use sulphate as a terminal electron acceptor to form hydrogen sulphide. Hydrogen sulphide (H_2_S) serves as a gasotransmitter in the maintenance of tissues homeostasis. It also has versatile effects in vasodilation, neuromodulation and anti-inflammation [41, 42]. *Coriobacteriaceae* plays a key role in the succession of gut microbial consortia in early life in humans [43]. The present study found that these beneficial bacteria were abundant in the appendix, indicating that the appendix may serve as a commensal pool that preserves a mass of probiotic bacteria to maintain the balance of the intestinal ecosystem. When the intestine was disordered, the flora of the jejunum became diverse, and the number of site-specific clusters increased. However, site-specific bacterial clusters were not identified in the disordered appendix. This finding is possibly due to the protective role of appendix biofilms with overwhelming commensal bacteria. The biofilm forms on the layer of mucus that covers the intestinal epithelium. The mucus consists of mucins rich in fucose, galactose, sialic acid, N-acetylgalactosamine, N-acetylglucosamine and mannose, some of which are produced by *Bacillus mesentericus* TO-A. The bacteria in the biofilm produce various producer-derived glycoside hydrolases to establish a metabolic interaction network that favours the growth of organisms that need carbon sources [31].

Former workers proposed a number of bacterial taxa in association with appendicitis in literature [13, 26–29]. We examined the distribution of these appendicitis-associated taxa in the jejunum, appendix and colon, and found that most appendicitis-associated taxa were detected in low level in our samples of both groups, except *Firmicutes* and *Bacteroidetes*. Salo et al. recovered *Firmicutes* in high level in the appendix of the patients with appendicitis, which agrees with our results. Toon Peeters et al. reported that the richness and diversity within the phyla *Firmicutes*, *Actinobacteria*, *Fusobacteria* and *Verrucomicrobia* were lower in faeces from those with appendicitis than in normal faeces [14]. Zhong D et al. recovered *Bacteroides* in high level in the normal appendix and the appendix with gangrenous appendicitis and they suggested the abundance of *Bacteroides* was inversely related to the degree of inflammation. Some scholars demonstrated that bacteria could migrate along the intestinal tract during appendicitis [44, 45]. Combining the findings from our study and from the others, we assume that the *Bacteroides* translocate retrogradely from the appendix and colon to the jejunum after antibiotic disruption. *Bacteroides* displacement might be both an outcome and a risk factor for inflammation progression to the appendix. With multicompartment profiling of the appendix-associated bacteria, we find no correlation between the appendicitis-associated taxa and the rat disordered appendix. Researchers have suggested that the excessive growth of appendiceal bacteria is a consequence of appendix obstruction and inflammation [12, 46], for example, *Fusobacterium* was present in most appendicitis specimens [47], and the number of species increased in parallel with the severity of the disease. In the present study, *Fusobacterium,* which was not present in the healthy gut, emerged in the dysbiosis jejunum. However, one should keep in mind that the appendix-associated taxa have been selected from the human with the onset of appendicitis.

Our antibiotic-induced dysbiosis model reveals that the ecological imbalance of the jejunum, appendix and colon likely link to gut inflammation and impairment. We found that the mucosal morphology changed apparently in the jejunum and colon but slightly in the appendix in the early stage of appendiceal inflammation. This result might be due to the protective role of the appendix. On the one hand, masses of lymphoid tissues are present in the appendix. On the other hand, appendix biofilms preserve high levels of gut probiotics to maintain community equilibrium. There are several ways of maintaining the integrity of the epithelial barrier and host microbial homeostasis in the gut. Intestinal goblet cells secrete mucin proteins acting as protective coatings that provide structural integrity and regulate macrophage and adaptive T cell responses during inflammatory processes [48]. Gastrointestinal dysfunction is associated with defects in the intestinal mucous lining caused by biotic or abiotic factors. This study characterized the gross morphology, mucosal morphology and cell structure of intestinal segments with light microscopy and electron transmission microscopy. Microvilli, brush-like edges, are the free surface of small intestinal epithelial cells. They are small finger-like protrusions that protrude from fibres in the cell membrane and cytoplasm. The function of microvilli is to expand the surface area of the free cells to enhance nutrient absorption. Microscopy revealed that the jejunal villus epithelial cells were impaired, the brush border was unclear, and mitochondria became swollen from the experiment group. Moreover, the abundance of microbiota such as *Betaproteobacteria*, *Pseudonocardiaceae*, *Enterococcaceae*, *Prevotellaceae* and *Enterococcus durans* changed in the experiment jejunum. We observed an inflated appendix with increasing fluids in the dysbiosis rats. Microscopy revealed that the appendix microvilli became sparse and broken. The local lymph nodules of the experiment appendix proliferated obviously, and the adjacent fibrous connective tissue also proliferated. These mucosal defects implicated that the appendix proceeded to early appendiceal inflammation. The role of dysbiotic microbiota in the development of appendiceal inflammation needs to be explored further. In the experiment colon, the epithelial cells were swollen and oedematous, and the microvilli were sparse. The microbiota richness and abundance reduced in the colon of the experiment animals. Microbiota imbalance or even loss of the colonial microflora leads to the exposure of microvilli to ceftriaxone sodium, which leads to the shrinkage of the villi, a reduction in absorption ability, and the accumulation of harmful substances. Microbiome functional profiling reveals the 20 functional pathways with the highest significant difference in the jejunum, appendix, and colon between the two groups. Most of these functional pathways were enriched in the control group regardless of the sites. The experiment appendix and colon shared two identical pathways, tetracycline biosynthesis and valine, leucine, and isoleucine degradation. Oxidative phosphorylation, which was exclusively enriched in the disordered appendix, is a metabolic pathway by which bacteria require oxygen to produce energy for cell survival. Arachidonic acid metabolism pathway, which was enriched in the disordered jejunum, has been indicated in association with inflammatory response [49].

To date, there has been a steady rise in studies of human microecology in association with health and disease. The link between gut intestinal dysbiosis and necrotizing enterocolitis has drawn great attention of researchers. The gut microbiota contributes to ecosystem equilibrium by increasing metabolic capacity, preventing the colonization of pathogenic organisms, providing vitamins and regulating the host immune system in human health [50]. Antibiotics have a comprehensive impact on the microbial community, which not only disrupts microbial survival but also alters the nutritional and pathophysiological status of the intestine.

## Conclusion

The present study investigated the microbiota composition, diversity and relative abundance, as well as mucosal morphology from the different intestinal segments of rats with/without antibiotic interruption. The microbiome species richness and evenness were high in the healthy appendix and colon but low in the disordered appendix and colon; the microbiome structure and relative abundance varied from the site to site inter- and intragroup; the appendix and colon shared similar microbiome patterns regardless of dysbiosis; and site-specific bacteria were missing in the disordered appendices. We find that appendix is likely a transition region involving in upper and lower intestinal microflora modulation. The present study reveals the in situ variation of gut microbiota under dysbiosis, which lays the foundation for exploring the microbial aetiology of diseases. However, we should be cautious about translating the microbiome profiling results from rat to human. Further research is needed on the human gut microbiota in the different stages of dysbiosis, as well as therapeutic effects of potential probiotics.

## Data Availability

Raw sequencing data are available in the NCBI Sequence Read Archive (https://www.ncbi.nlm.nih.gov/sra) under study accession PRJNA594467.

## References

[1] Tropini C, Earle KA, Huang KC, Sonnenburg JL (2017). The gut microbiome: connecting spatial organization to function. Cell Host Microbe.

[2] Ley RE, Bäckhed F, Turnbaugh P, Lozupone CA, Knight RD, Gordon JI (2005). Obesity alters gut microbial ecology. Proc Natl Acad Sci USA.

[3] Vyas U, Ranganathan N (2012). Probiotics, prebiotics, and synbiotics: gut and beyond. Gastroenterol Res Pract.

[4] Cummings JH, Pomare EW, Branch WJ, Naylor CP, Macfarlane GT (1987). Short chain fatty acids in human large intestine, portal, hepatic and venous blood. Gut.

[5] Guarner F, Malagelada JR (2003). Gut flora in health and disease. Lancet.

[6] Gomaa EZ (2020). Human gut microbiota/microbiome in health and diseases: a review. Antonie Van Leeuwenhoek.

[7] Bollinger R, Barbas AS, Bush EL, Lin SS, Parker W (2007). Biofilms in the large bowel suggest an apparent function of the human vermiform appendix. J Theor Biol.

[8] Laurin M, Everett ML, Parker W (2011). The cecal appendix: one more immune component with a function disturbed by post-industrial culture. Anat Rec (Hoboken).

[9] Guillet-Caruba C, Cheikhelard A, Guillet M, Bille E, Descamps P, Yin L, Khen-Dunlop N, Zahar JR, Sarnacki S, Revillon Y (2011). Bacteriologic epidemiology and empirical treatment of pediatric complicated appendicitis. Diagn Microbiol Infect Dis.

[10] Chen CY, Chen YC, Pu HN, Tsai CH, Chen WT, Lin CH (2012). Bacteriology of acute appendicitis and its implication for the use of prophylactic antibiotics. Surg Infect (Larchmt).

[11] Roberts JP (1988). Quantitative bacterial flora of acute appendicitis. Arch Dis Child.

[12] Guinane CM, Tadrous A, Fouhy F, Ryan CA, Dempsey EM, Murphy B, Andrews E, Cotter PD, Stanton C, Ross RP (2013). Microbial composition of human appendices from patients following appendectomy. MBio.

[13] Zhong D, Brower-Sinning R, Firek B, Morowitz MJ (2014). Acute appendicitis in children is associated with an abundance of bacteria from the phylum Fusobacteria. J Pediatr Surg.

[14] Peeters T, Penders J, Smeekens SP, Galazzo G, Houben B, Netea MG, Savelkoul PH, Gyssens IC (2019). The fecal and mucosal microbiome in acute appendicitis patients: an observational study. Future Microbiol.

[15] Yin J, Prabhakar M, Wang S, Liao SX, Peng X, He Y, Chen YR, Shen HF, Su J, Chen Y (2015). Different dynamic patterns of beta-lactams, quinolones, glycopeptides and macrolides on mouse gut microbial diversity. PLoS One.

[16] Bobin-Dubigeon C, Collin X, Grimaud N, Robert JM, Le Baut G, Petit JY (2001). Effects of tumour necrosis factor-alpha synthesis inhibitors on rat trinitrobenzene sulphonic acid-induced chronic colitis. Eur J Pharmacol.

[17] Sundberg C, Al-Soud WA, Larsson M, Alm E, Yekta SS, Svensson BH, Sorensen SJ, Karlsson A (2013). 454 pyrosequencing analyses of bacterial and archaeal richness in 21 full-scale biogas digesters. FEMS Microbiol Ecol.

[18] Martin M (2011). CUTADAPT removes adapter sequences from high-throughput sequencing reads. EMBnetjournal.

[19] Haas BJ, Gevers D, Earl AM, Feldgarden M, Ward DV, Giannoukos G, Ciulla D, Tabbaa D, Highlander SK, Sodergren E (2011). Chimeric 16S rRNA sequence formation and detection in Sanger and 454-pyrosequenced PCR amplicons. Genome Res.

[20] Edgar RC, Haas BJ, Clemente JC, Quince C, Knight R (2011). UCHIME improves sensitivity and speed of chimera detection. Bioinformatics.

[21] Edgar RC (2013). UPARSE: highly accurate OTU sequences from microbial amplicon reads. Nat Methods.

[22] Wang Q, Garrity GM, Tiedje JM, Cole JR (2007). Naive Bayesian classifier for rapid assignment of rRNA sequences into the new bacterial taxonomy. Appl Environ Microbiol.

[23] Quast C, Pruesse E, Yilmaz P, Gerken J, Schweer T, Yarza P, Peplies J, Glockner FO (2013). The SILVA ribosomal RNA gene database project: improved data processing and web-based tools. Nucleic Acids Res.

[24] Edgar RC (2004). MUSCLE: multiple sequence alignment with high accuracy and high throughput. Nucleic Acids Res.

[25] Segata N, Izard J, Waldron L, Gevers D, Miropolsky L, Garrett WS, Huttenhower C (2011). Metagenomic biomarker discovery and explanation. Genome Biol.

[26] Salo M, Marungruang N, Roth B, Sundberg T, Stenstrom P, Arnbjornsson E, Fak F, Ohlsson B (2017). Evaluation of the microbiome in children's appendicitis. Int J Colorectal Dis.

[27] Wagner M, Tubre DJ, Asensio JA (2018). Evolution and current trends in the management of acute appendicitis. Surg Clin North Am.

[28] Schulin S, Schlichting N, Blod C, Opitz S, Suttkus A, Stingu CS, Barry K, Lacher M, Buhligen U, Mayer S (2017). The intra- and extraluminal appendiceal microbiome in pediatric patients: A comparative study. Medicine (Baltimore).

[29] Alkadhi S, Kunde D, Cheluvappa R, Randall-Demllo S, Eri R (2014). The murine appendiceal microbiome is altered in spontaneous colitis and its pathological progression. Gut Pathog.

[30] Lamps LW (2010). Infectious causes of appendicitis. Infect Dis Clin North Am.

[31] Rakoff-Nahoum S, Coyne MJ, Comstock LE (2014). An ecological network of polysaccharide utilization among human intestinal symbionts. Curr Biol.

[32] Backhed F, Ley RE, Sonnenburg JL, Peterson DA, Gordon JI (2005). Host-bacterial mutualism in the human intestine. Science.

[33] Xu J, Bjursell MK, Himrod J, Deng S, Carmichael LK, Chiang HC, Hooper LV, Gordon JI (2003). A genomic view of the human-Bacteroides thetaiotaomicron symbiosis. Science.

[34] Riva A, Borgo F, Lassandro C, Verduci E, Morace G, Borghi E, Berry D (2017). Pediatric obesity is associated with an altered gut microbiota and discordant shifts in Firmicutes populations. Environ Microbiol.

[35] Schade J, Weidenmaier C (2016). Cell wall glycopolymers of Firmicutes and their role as nonprotein adhesins. FEBS Lett.

[36] Lucas M (1983). Determination of acid surface pH in vivo in rat proximal jejunum. Gut.

[37] Al Kassaa I, Hober D, Hamze M, Chihib NE, Drider D (2014). Antiviral potential of lactic acid bacteria and their bacteriocins. Probiotics Antimicrob Proteins.

[38] Kameyama K, Itoh K (2014). Intestinal colonization by a Lachnospiraceae bacterium contributes to the development of diabetes in obese mice. Microbes Environ.

[39] Donaldson GP, Lee SM, Mazmanian SK (2016). Gut biogeography of the bacterial microbiota. Nat Rev Microbiol.

[40] Shang Q, Shan X, Cai C, Hao J, Li G, Yu G (2016). Dietary fucoidan modulates the gut microbiota in mice by increasing the abundance of Lactobacillus and Ruminococcaceae. Food Funct.

[41] Zhang-Sun W, Augusto LA, Zhao L, Caroff M (2015). Desulfovibrio desulfuricans isolates from the gut of a single individual: structural and biological lipid A characterization. FEBS Lett.

[42] Magierowski M, Jasnos K, Kwiecien S, Drozdowicz D, Surmiak M, Strzalka M, Ptak-Belowska A, Wallace JL, Brzozowski T (2015). Endogenous prostaglandins and afferent sensory nerves in gastroprotective effect of hydrogen sulfide against stress-induced gastric lesions. PLoS One.

[43] Harmsen HJ, Wildeboer-Veloo AC, Grijpstra J, Knol J, Degener JE, Welling GW (2000). Development of 16S rRNA-based probes for the Coriobacterium group and the Atopobium cluster and their application for enumeration of Coriobacteriaceae in human feces from volunteers of different age groups. Appl Environ Microbiol.

[44] Coldewey SM, Hartmann M, Schmidt DS, Engelking U, Ukena SN, Gunzer F (2007). Impact of the rpoS genotype for acid resistance patterns of pathogenic and probiotic Escherichia coli. BMC Microbiol.

[45] Blod C, Schlichting N, Schulin S, Suttkus A, Peukert N, Stingu CS, Hirsch C, Elger W, Lacher M, Buhligen U (2018). The oral microbiome-the relevant reservoir for acute pediatric appendicitis?. Int J Colorectal Dis.

[46] Wangensteen OH, Dennis C (1939). Experimental proof of the obstructive origin of appendicitis in man. Ann Surg.

[47] Swidsinski A, Dorffel Y, Loening-Baucke V, Theissig F, Ruckert JC, Ismail M, Rau WA, Gaschler D, Weizenegger M, Kuhn S (2011). Acute appendicitis is characterised by local invasion with Fusobacterium nucleatum/necrophorum. Gut.

[48] Robinson K, Deng Z, Hou Y, Zhang G (2015). Regulation of the intestinal barrier function by host defense peptides. Front Vet Sci.

[49] Powell WS, Rokach J (1851). 2015 Biosynthesis, biological effects, and receptors of hydroxyeicosatetraenoic acids (HETEs) and oxoeicosatetraenoic acids (oxo-ETEs) derived from arachidonic acid. Biochim Biophys Acta.

[50] Stein RR, Bucci V, Toussaint NC, Buffie CG, Ratsch G, Pamer EG, Sander C, Xavier JB (2013). Ecological modeling from time-series inference: insight into dynamics and stability of intestinal microbiota. PLoS Comput Biol.

